# To Lead or to Follow: Contribution of the Plant Vacuole to Cell Growth

**DOI:** 10.3389/fpls.2020.00553

**Published:** 2020-05-08

**Authors:** Sabrina Kaiser, David Scheuring

**Affiliations:** Plant Pathology, University of Kaiserslautern, Kaiserslautern, Germany

**Keywords:** vacuole, cell elongation, auxin, cell wall, turgor, cell size, cytoskeleton, actin

## Abstract

Cell division and cell elongation are fundamental processes for growth. In contrast to animal cells, plant cells are surrounded by rigid walls and therefore loosening of the wall is required during elongation. On the other hand, vacuole size has been shown to correlate with cell size and inhibition of vacuolar expansion limits cell growth. However, the specific role of the vacuole during cell elongation is still not fully resolved. Especially the question whether the vacuole is the leading unit during cellular growth or just passively expands upon water uptake remains to be answered. Here, we review recent findings about the contribution of the vacuole to cell elongation. In addition, we also discuss the connection between cell wall status and vacuolar morphology. In particular, we focus on the question whether vacuolar size is dictated by cell size or *vice versa* and share our personnel view about the sequential steps during cell elongation.

## Introduction

The plants largest organelle, the vacuole, occupies up to 90% of the cellular volume in vegetative tissues. Although the name *vacuole* originates from the Latin *vacuus* (= vacuum), implying an empty and potentially functionless space, quite the opposite is true. Vacuoles fulfill a plethora of important and diverse functions in plant cells. Among them are the degradation of cellular waste, the storage of ions and proteins, plant defense against pathogens, pH homeostasis and plant growth. The vacuole’s prominent size and the finding that individual cells hold turgor pressure up to 5 bar (Zimmermann et al., 1980) established the believe that vacuoles provide the driving force for plant growth. Here, vacuoles were thought to simply drive cell elongation via turgor pressure (Marty, 1999). While there is no doubt that turgor pressure contributes to cell elongation (Cosgrove, 2018), the precise role of the vacuole in this process remains unclear and has not yet been addressed experimentally. However, restricting the vacuole’s dimensions has been shown to inhibit cellular elongation and root organ growth (Löfke et al., 2015; Kaiser et al., 2019). Conversely, increasing vacuolar occupancy of the cell is of eminent importance during elongation (Dünser et al., 2019).

Besides the vacuole, the properties of the plant cell wall represent another crucial component for defining cell size and growth rates. Cell wall acidification and subsequent loosening are long known to be a prerequisite for expansion (Cosgrove, 1993), but the interplay with intracellular changes (vacuolar expansion) are less well understood. Only lately substantial progress was made in understanding the signaling and coordination between extracellular and intracellular changes during cell elongation (Dünser et al., 2019).

## Cell Wall Loosening as Prerequisite for Cell Elongation

Since plant cells are sheathed by rigid, shape-giving cell walls, cellular extension cannot be explained by vacuolar expansion alone but must, consequently, also include cell wall modifications. Already in the early 70s of the last century, researchers could show that the elongation of stem and coleoptile cells promoted by the phytohormone auxin was coupled to cell wall loosening (Rayle and Cleland, 1970; Hager et al., 1971). This led to the formulation of the so-called *acid growth theory*, primarily proposing a connection of auxin and acidification of the cell exterior, the apoplast. The current understanding of this hypothesis includes the activation of plasma membrane (PM) H^+^-ATPases by auxin and subsequent acidification of the apoplast and thus the cell wall. Following this, pH-responsive non-enzymatic proteins from the expansin family (McQueen-Mason et al., 1992) are activated, leading to cell wall loosening via xyloglucan slipping (Cosgrove, 2000). Cell elongation is then achieved by water uptake alongside with the deposition of new wall material.

Not long ago, the acid growth theory has been confirmed in shoots (Fendrych et al., 2016) and roots of the model plant Arabidopsis (Barbez et al., 2017). In the latter, the model was heavily debated, due to the complex role of auxin in plant development and technical limitations in investigating apoplastic pH at cellular resolution. Depending on the concentration and the cell type, auxin promotes and inhibits growth, respectively. In the physiological concentration range, auxin preferentially induces growth in aerial, and inhibits growth in underground tissues (Evans et al., 1994; Dünser and Kleine-Vehn, 2015). However, Barbez and coauthors showed that cell wall acidification induced cellular expansion and that this is preceded by and dependent on auxin signaling (Barbez et al., 2017). Interestingly, increasing total levels of auxin induced a transient alkalinization of the apoplast and reduced cellular elongation. This in turn was dependent on the receptor-like kinase FERONIA (FER) which has been demonstrated to control the elasticity of the cell wall (Höfte, 2015) and functions as a mechano−sensor (Shih et al., 2014).

## Contribution of the Vacuole to Cell Elongation

It has been shown that auxin does not only affect cell wall loosening but also directly impacts on the vacuole. In the Arabidopsis root meristem, the phytohormone induced smaller and more constricted vacuoles (Löfke et al., 2015). Changes of vacuolar morphology in turn directly affected cell-size control and restricted root growth (Löfke et al., 2015). Since this auxin-induced vacuolar phenotype was accompanied by an increased abundance of soluble N-ethylmaleimide-sensitive-factor attachment receptors (SNAREs), especially VTI11, auxin was hypothesized to impact on homotypic vacuolar fusion events (Löfke et al., 2015). Subsequently, auxin-induced constrictions of the vacuole were demonstrated to be dependent on the actin cytoskeleton (Scheuring et al., 2016). In actin and myosin mutants, auxin-induced changes of vacuolar morphology, cell-size restriction and inhibition of root growth were all largely abolished (Scheuring et al., 2016). In mammalian cells, members of the HOPS (homotypic fusion and protein sorting) complex interact with the actin cytoskeleton (Richardson et al., 2004). This complex mediates homotypic vacuole fusion in plants (Takemoto et al., 2018) and a potential actin interaction here would at least partly explain the actin-dependency of auxin-induced vacuolar changes.

Notably, the tonoplast-localized auxin transporter WALLS ARE THIN1 1 (WAT1) has been identified, facilitating auxin export from the vacuole (Ranocha et al., 2013). Hence, a key role for the vacuole in intracellular auxin homeostasis was suggested. Moreover, WAT1 has been proposed to integrate auxin signaling and secondary cell wall formation of stem fibers in Arabidopsis (Ranocha et al., 2010).

Another layer of complexity is added by the presence of different vacuole types in plants. Dependent on their function, vacuoles are classified into protein storage vacuoles (PSVs), predominantly found in seed tissues, and lytic vacuoles (LVs) which are commonly found in vegetative tissue and are primarily discussed in the present mini-review. For both, PSVs and LVs, independent inhibition of trafficking has been demonstrated (Bolte et al., 2004; Park et al., 2005), and thus separate transport routes were assumed. Furthermore, it has been shown that trafficking to these vacuoles requires different members of SNARE proteins. While VTI12 plays an important role in protein transport to PSVs, trafficking to the LV is mainly dependent on VTI11 (Sanmartín et al., 2007). This could indicate that the auxin induced vacuolar changes seen for LVs accompanied by upregulation of VTI11 (Löfke et al., 2015) are unique for LVs, potentially not impacting on PSVs.

To unravel the morphological changes of the lytic vacuole upon exogenous auxin treatment in detail, the combination of state-of-the-art imaging and staining methods were employed. This allowed 3D modeling and quantitative analysis of different parameters such as vacuolar volume and surface area. Markedly, upon auxin application, most vacuolar subvolumina were still connected and together formed one single interconnected organelle (Scheuring et al., 2015, 2016). Based on the reduced cellular space that (auxin-induced) constricted vacuoles occupy, a space-filling function of the vacuole was proposed. This would allow plant cells to elongate without altering the amount of cytosol, thereby massively reducing energy investment (Scheuring et al., 2016; Krüger and Schumacher, 2018). Accordingly, auxin would limit the intracellular occupancy of the vacuole to restrict cell elongation. This is well in accordance with the observation that the size of plant vacuoles correlates with cell size (Owens and Poole, 1979).

Due to the close proximity of the vacuolar membrane (tonoplast) and actin filaments it has been suggested that there might be a direct physical connection (Kutsuna et al., 2003). In a screen for GFP-fusion proteins labeling actin filaments, the plant-specific Networked (NET) family was identified (Deeks et al., 2012). NET proteins possess an actin-binding domain and are membrane-associated, thus linking actin filaments with different cell organelles. One specific member, NET4A, has been shown to bind actin and overlaps with the tonoplast (Deeks et al., 2012). Recently, it was shown that NET4A localizes to highly constricted regions of the tonoplast and, together with NET4B, modulates vacuolar occupancy. Overexpression led to a decrease, and loss-of-function to an increase of vacuolar occupancy, respectively (Kaiser et al., 2019). As increased vacuolar volume allows for rapid cellular elongation with relatively little *de novo* production of cytosolic content (Dünser et al., 2019); more compact vacuoles induced by NET4A overexpression might explain the accompanied cell size and root length limitations observed in a NET4A overexpressor line (Kaiser et al., 2019). Furthermore, this finding confirms that cell size and vacuole size are tightly linked and that inhibited cell elongation via restricted vacuolar size does not exclusively depend on auxin. Indeed, additional factors, such as blue light and most of the other described phytohormones are also involved in cell size determination (Halliday et al., 2009; Perrot-Rechenmann, 2010).

Naturally, the observed restrictions of vacuolar expansion led to the question of their function apart from restricting cell size. One explanation could be the requirement of increasing cytosol demands during cell division. In agreement with this, vacuolar volume has been reported to decrease by 80% within cell division, increasing the amount of cytosol required to accommodate the forming phragmoplast and associated cell plate-forming structures (Seguí-Simarro and Staehelin, 2006). In line with this, unequal vacuole partitioning during embryogenesis in the *gravitropism defective 2* (grv2) mutant resulted in daughter cell formation of unequal size (Silady et al., 2008). It was postulated that the presence of an unusually large vacuole in one daughter cell led to misalignment of the phragmoplast, explaining the observed disturbance of cell division in the mutant (Silady et al., 2008).

**FIGURE 1:**
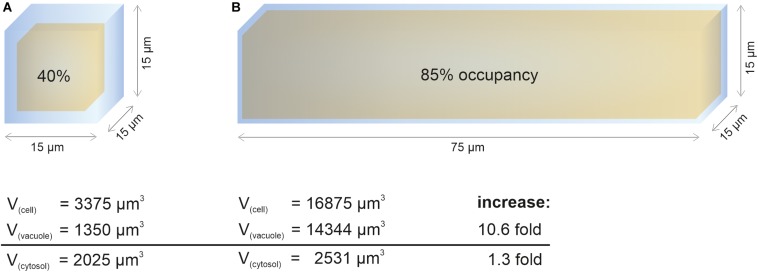
Cytosol homeostasis during cell expansion. The cellular space occupied by the vacuole (occupancy) increases dramatically during elongation, allowing to keep the cytosolic volume relatively constant. For simplification, the geometric body of a cube was used to calculate vacuolar volume (yellow) and cell volume (blue). After subtraction, the remaining space was used to approximate cytosolic volume. Values for vacuolar occupancy were based on Dünser et al. (2019) in this figure. **(A)** Simplified epidermis cell of the late meristem from the Arabidopsis root with the edge length of 15 μm. **(B)** Simplified 5-fold elongated cell from the transition zone. While the vacuolar volume increases more than 10-fold during elongation, the cytosolic volume only increases by factor 1.3.

In contrast to vacuole size restriction during cytokinesis, developing cells of the root meristem display gradually larger vacuoles as cells transit into the elongation zone. Cells leaving meristematic zones extend their original size 10–1,000-fold (Veytsman and Cosgrove, 1998). To fulfill its space-filling function and to avoid high metabolic costs for the generation of large amounts of cytosolic content, the vacuole must dramatically increase its volume (Figure 1). Indeed, vacuolar occupancy of the cell increases from around 40% in meristematic cells to more than 85% in cells of the late elongation zone (Dünser et al., 2019). Initially, the osmotic potential in the vacuole must be higher than in the cytosol to enable water uptake, but eventually it must reach equilibrium. The tonoplast (unlike the cell wall) has limited tensile strength and cannot withstand significant differences in pressure without rupturing.

## Discussion

Obviously, vacuolar expansion and cell wall loosening both are of eminent importance for cell elongation. Thus, the question arises: How are these processes coordinated and is the vacuole indeed the driving force for cell elongation as proposed previously?

Recent data revealed that the receptor-like kinase FER together with extracellular leucine−rich repeat extensins (LRXs) sense cell wall properties (such as loosening) and subsequently impact on the intracellular expansion of the vacuole (Dünser et al., 2019). This module was proposed to integrate the cell wall status with intracellular growth processes, but it remained unclear how precisely LRX/FER signaling at the cell surface leads to the modulation of vacuolar size. One hypothesis involves several transduction steps to regulate the actin cytoskeleton (Dünser et al., 2019). Since the actin cytoskeleton surrounds the vacuole and contributes to the regulation of vacuolar size (Scheuring et al., 2016), this link could in principle explain how extracellular sensing and intracellular control of vacuolar volume are integrated. In agreement, *lrx* and *fer* mutants display a pronounced enlargement of the vacuolar lumina and a higher vacuolar occupancy of the cell (Dünser et al., 2019). Moreover, these vacuoles are resistant to pharmacological treatments that presumably impact on cell wall properties and normally restrict vacuolar expansion (Dünser et al., 2019). Thus, it is tempting to state that without transmission of the cell wall status, vacuolar changes will not occur. Additionally, in fully elongated cells, the osmotic potential of cytosol and vacuole needs to be equilibrated to prevent membrane rupture. It is conceivable that a high turgor pressure could be build up without the vacuole, albeit with dramatically higher energy investment.

Furthermore, it has been shown that the expression of a specific tonoplast intrinsic protein (γ-TIP), enabling water uptake, is correlated with cell elongation (Ludevid et al., 1992). If the vacuole would be indeed the driving force, one would expect the onset of TIP expression before cell elongation is initiated. In contrast, several TIPs were found to be preferentially expressed in elongating cells but not in the meristematic zone of Arabidopsis roots (Gattolin et al., 2009, 2010). This again questions the vacuole’s role in leading cell expansion via increasing turgor pressure.

**FIGURE 2:**
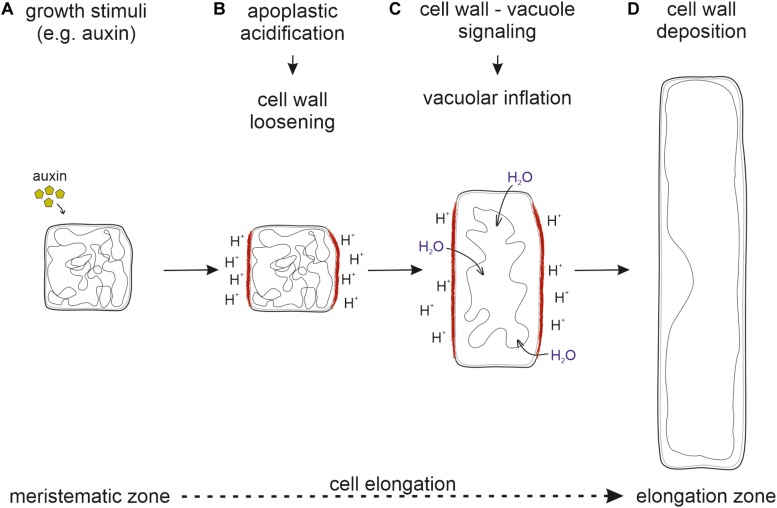
Sequential order of processes necessary for cell elongation. **(A)** Upon perception of growth stimuli, **(B)** the apoplast is acidified and the cell wall loosened. **(C)** The cell wall status is sensed by the FER-LRX module and transmitted into the cell. **(D)** New cell wall material deposition allows for cell elongation. Inhibition at any step prevents proper cell elongation and inhibits growth.

Taken together, it seems likely that cell wall loosening through apoplastic acidification is the driving force for vacuolar expansion. For this, the cell wall status has to be sensed and external signals must be transmitted into the cell to initiate vacuolar expansion. Only then, the space-filling capacity of the vacuole allows the occupation of the emerging space in expanding cells. This ensures that cytosolic content does not become the growth-limiting factor (Figure 2).

Therefore, we believe that vacuolar expansion follows cell wall loosening which marks the onset for cell expansion. Only in concert, both processes jointly grant rapid cell elongation and enable fast plant growth rates. However, the precise relationship between vacuole expansion, cell wall properties and cell elongation is not fully understood and it will be an exciting future task to identify yet unknown players involved in the coordination of this complex and delicate process.

Another important question that needs to be addressed in future research is the source of membrane material to allow for rapid cell elongation. Due to the enormous increase in cell size, not only the vacuole but also the PM requires new membrane material to adapt their surface area. While the endoplasmic reticulum (ER) is the main synthesis site for lipids destined for the PM (Blom et al., 2011) the source for newly synthesized vacuole membrane is not yet unanimously agreed on. During vacuole biogenesis two seemingly opposing models are controversially discussed. One describes the ER as the main membrane source while the second considers small vacuoles (SV), derived through fusion and maturing of multivesicular bodies (MVBs) which in turn originate from the trans-Golgi network (TGN) as membrane carrier (reviewed in Viotti et al., 2013; Krüger and Schumacher, 2018; Cui et al., 2020). Certainly, rearrangement of the convoluted and constricted tonoplast in meristematic cells will provide some excessive membrane material for gradually expanding vacuoles (Figure 2), but this seems hardly sufficient for full expansion in elongated cells. Therefore, it must not only shed light upon the relationship between vacuole expansion and cell wall properties, but also investigated how membrane material is delivered to the PM and the tonoplast during cell elongation. In addition, the spatiotemporal coordination of cell wall loosening and vacuole expansion with growing PM surface area remains to be addressed. Understanding the integration of all these processes and the sequential order of events will provide challenging future research tasks.

## Author Contributions

SK and DS wrote the manuscript and prepared the figures.

## Conflict of Interest

The authors declare that the research was conducted in the absence of any commercial or financial relationships that could be construed as a potential conflict of interest.
